# TELL 2.0: Interpretable speech biomarkers for dementia in research and clinical applications

**DOI:** 10.1002/alz70863_110670

**Published:** 2025-12-23

**Authors:** Franco Javier Ferrante, Gonzalo Nicolás Pérez, Joaquín Ponferrada, Alejandro Sosa Welford, Nicolás Pelella, Matías Caccia, Laouen Belloli, Cecilia Calcaterra, Catalina González Santibáñez, Raúl Echegoyen, Mariano Javier Cerruti, Fernando Johann, Eugenia Hesse, Facundo Carrillo, Adolfo M Garcia

**Affiliations:** ^1^ Consejo Nacional de Investigaciones Científicas y Técnicas (CONICET), Buenos Aires Argentina; ^2^ Cognitive Neuroscience Center, University of San Andrés, Victoria, Buenos Aires Argentina; ^3^ Universidad de Buenos Aires, Buenos Aires Argentina; ^4^ Cognitive Neuroscience Centre, CABA, Buenos Aires Argentina; ^5^ University of Buenos Aires, Ciudad Autonoma de Buenos Aires, CABA Argentina; ^6^ Cognitive Neuroscience Center, CABA, Buenos Aires Argentina; ^7^ TELL Toolkit SA, Buenos Aires, Buenos Aires Argentina; ^8^ Departamento de Lingüística y Literatura, Facultad de Humanidades, Universidad de Santiago de Chile, Santiago de Chile, Santiago de Chile Argentina; ^9^ School of Engineering, ORT University, Montevideo, Montevideo Uruguay; ^10^ Cognitive Neuroscience Centre, University of San Andres, Victoria, Buenos Aires Argentina; ^11^ Universidad de San Andrés, Victoria, Buenos Aires Argentina; ^12^ Instituto de Ciencias de la Computación, CONICET‐UBA, CABA, Buenos Aires Argentina; ^13^ Cognitive Neuroscience Center (CNC), Universidad de San Andrés, Buenos Aires, Buenos Aires Argentina; ^14^ Faculty of Education, National University of Cuyo, Mendoza Argentina; ^15^ Universidad de Santiago de Chile, Santiago, Santiago Chile; ^16^ BrainLat Institute, Santiago, Santiago Chile; ^17^ INECO Foundation, Buenos Aires Argentina

## Abstract

**Background:**

Launched in early 2023, the Toolkit to Examine Lifelike Language (TELL) is a web app designed to gather voice samples and extract speech biomarkers for dementia. Its initial version enhanced clinical evaluations across diverse regions, including under‐resourced countries. Building on this success, we have developed TELL 2.0, which introduces advanced features for task delivery, data (pre)processing, analysis, and visualization.

**Method:**

TELL's advancements are driven by a multidisciplinary team of neurolinguists, data scientists, engineers, and programmers. Recent updates include significant backend and frontend improvements. First, the app's web‐based system for in‐person administration now supports videocall‐based and offline data collection, offering lossless audio files and transcriptions via a locally hosted version of OpenAI's Whisper model. Second, a comprehensive audio standardization pipeline has been added, including amplitude normalization, noise reduction, and channel harmonization. Third, new interpretable metrics validated by our latest research are integrated, covering lexical retrieval (e.g., pause durations), motor speech abilities (e.g., vocal‐fold activation efficiency), semantic memory patterns (e.g., semantic granularity, which reflects conceptual precision across discourse), and self‐referential language (via morphological tagging of grammatical person). Fourth, an intuitive visualization interface now compares individual results against normative data and provides machine learning‐based predictions of similarity to profiles typical of Alzheimer's disease, mild cognitive impairment, and behavioral variant frontotemporal dementia.

**Result:**

Testing demonstrates consistent audio and transcription quality regardless of the data collection mode. The new metrics distinguish individuals with specific disorders from healthy controls with accuracies ranging from 85 to 93%. These metrics align closely with established neuropsychological frameworks for each condition, optimizing their clinical relevance. Feedback highlights that the updated visualization interface provides clinicians with actionable insights.

**Conclusion:**

With its enhanced capabilities, TELL 2.0 broadens the accessibility and impact of speech biomarkers, improving on its predecessor. Our research‐driven approach focuses on transforming cutting‐edge findings into practical, scalable, and cost‐effective tools for dementia evaluation.

